# Clinicopathological Patterns and Predictors of the Functional Restoration of Immunoglobulin G4-Related Kidney Disease: A Chinese Single-Center Cohort Study

**DOI:** 10.3389/fmed.2021.736098

**Published:** 2021-10-06

**Authors:** Tao Su, Hui Wang, Suxia Wang, Li Yang

**Affiliations:** ^1^Department of Nephrology, Peking University First Hospital, Peking University Institute of Nephrology, Beijing, China; ^2^Renal Pathology Center, Institute of Nephrology, Peking University, Beijing, China; ^3^Laboratory of Electron Microscopy, Pathological Center, Peking University First Hospital, Beijing, China

**Keywords:** IgG4-related disease, kidney disease, tubulointerstitial nephritis, crescentic nephritis, IgG4 autoantibodies, membranous nephropathy, prognosis

## Abstract

**Background:** Immunoglobulin G4-related disease (IgG4-RD) is a systemic immunoreactivity-based fibro-inflammatory disease. Immunoglobulin G4-related kidney disease (IgG4-RKD) is a frequently overlooked diagnosis. This study aimed to describe IgG4-RKD and examine the factors relevant to the renal outcomes of IgG4-RD.

**Methods:** We studied a prospective IgG4-RKD cohort between January 2012 and December 2020 with close follow-up. Clinicopathologic data at kidney biopsy were collected and analyzed. We aimed to explore independent risk factors for long-term renal outcome and disease relapse. Patients with an eGFR<45 ml/min per 1.73m^2^ at 12 months were defined as having poor outcomes.

**Results:** The included 42 patients with IgG4-RKD had a mean age of 58.5 ± 8.7 years (male-to-female ratio = 5:1). The IgG4-RD responder index (RI) was 12.2 ± 3.3. A total of 66.7% of the patients presented with acute on kidney disease or acute on chronic kidney disease. Eight patients (19.0%) showed nephrotic-range proteinuria, and nine (21.4%) had high-titer IgG4-autoantibodies, including antineutrophil cytoplasmic antibody and anti-phospholipase A2 receptor. A kidney biopsy was conducted in 40 patients. Thirty-seven (90.0%) patients were diagnosed with IgG4-related tubulointerstitial nephritis, and 19 (47.5%) of them had concurrent glomerular diseases (membranous nephropathy [MN], *n* = 3; crescentic glomerulonephritis [CrGN], *n* = 11; diabetic kidney disease, *n* = 3; and both MN and CrGN, *n* = 2). IgG4-RD RI had a close relationship with serum C3 (*R* = −0.509, *P* = 0.001), C4 (*R* = −0.314, *P* = 0.049) levels, and peripheral blood eosinophil count (PBEC; *R* = 0.377, *P* = 0.024), factors that were not included in RI scores. Correlation analysis disclosed that IgG4-RD RI (*R* = 0.422, *P* = 0.007), organs involved (*R* = 0.452, *P* = 0.003), and C3 (*R* = −0.487, *R* = 0.002) were correlated with the percentage decrease of serum creatinine at 1 month. However, multivariate regression analysis failed to identify any clinicopathological parameters that could predict short-term renal restoration and IgG4-RKD relapse. Ten out of 29 variables, of most importance, were identified by the least absolute shrinkage and selection operator (LASSO) regression analysis. By multivariate logistic regression a higher serum IgG4 (OR = 0.671, *P* = 0.010), IgG1 (OR = 1.396, *P* = 0.049), IgG3 (OR = 19.154, *P* = 0.039), and erythrocyte sedimentation rate (ESR; OR = 1.042, *P* = 0.032) were found to be independent factors for poor long-term outcome. Conventional immunosuppressive medications and/or rituximab were prescribed, and in 83.3% of the patients, the kidney function improved. Repeat kidney biopsies confirmed the remission of interstitial inflammation in two patients under immunosuppressive therapy. However, the disease relapse rate was as high as 31.0%.

**Conclusions:** We strongly recommend a kidney biopsy in active IgG4-RD, especially when there is proteinuria and renal dysfunction, because concurrent glomerular involvement and active interstitial inflammation should be assessed. A higher serum IgG1, IgG3, and ESR were independent factors for the poor long-term renal outcome; however, elevated IgG4 predicted a good renal prognosis, and appropriate and timely immunosuppressive therapy can help achieve a better prognosis.

## Introduction

Immunoglobulin G4-related disease (IgG4-RD) is characterized by immunoreactivity-based fibro-inflammation and multiple-organ involvement (including the kidney). IgG4-related kidney disease (IgG4-RKD) represents any disease with renal involvement by IgG4-RD (1, 2). The prevalence of IgG4-RKD is reported to be 6.9–27.4% (1, 3, 4). IgG4-RKD has various clinical manifestations, including inflammatory pseudo-tumors, impaired renal function, nephrotic syndrome, and urinary tract obstruction induced by IgG4-related pyelitis or retroperitoneal fibrosis. Currently, the diagnosis of IgG4-RD is based on the diagnostic and classification criteria proposed by the Japanese Rheumatic Association (2011) and the American Rheumatic Association/European Rheumatic Alliance (2019), respectively (5–8). The diagnosis of IgG4-RKD is also based on the criteria defined by these associations and on characteristic renal imaging findings and pathological features (2). A fibro-inflammatory lesion with IgG4-rich plasma cell tissue infiltration is the characteristic histopathological change. In addition, the pathogenesis of IgG4-RD is based on an autoimmune response; however, it mimics the presentation of other autoimmune diseases, which results in overlapping symptoms.

However, the renal outcomes of IgG4-RKD remain unexplained, and the assessment of the systemic inflammatory state in IgG4-RD with the IgG4-RD responder index (RI) inadequately quantifies the degree of renal inflammation (9). An improvement in the renal function in IgG4-RKD indicates that the disease is responsive to steroids; therefore, if the active inflammation can be detected earlier, the prognosis of kidney will be better improved and the transition from acute kidney injury (AKI) to severe chronic kidney disease (CKD) will be reduced. This study aimed to summarize the clinicopathological characteristics and factors relevant to the diagnosis and renal outcomes of patients with IgG4-RKD in a cohort of patients with biopsy-proven IgG4-RKD.

## Materials and Methods

### Study Design and Patients

**Figure 1:**
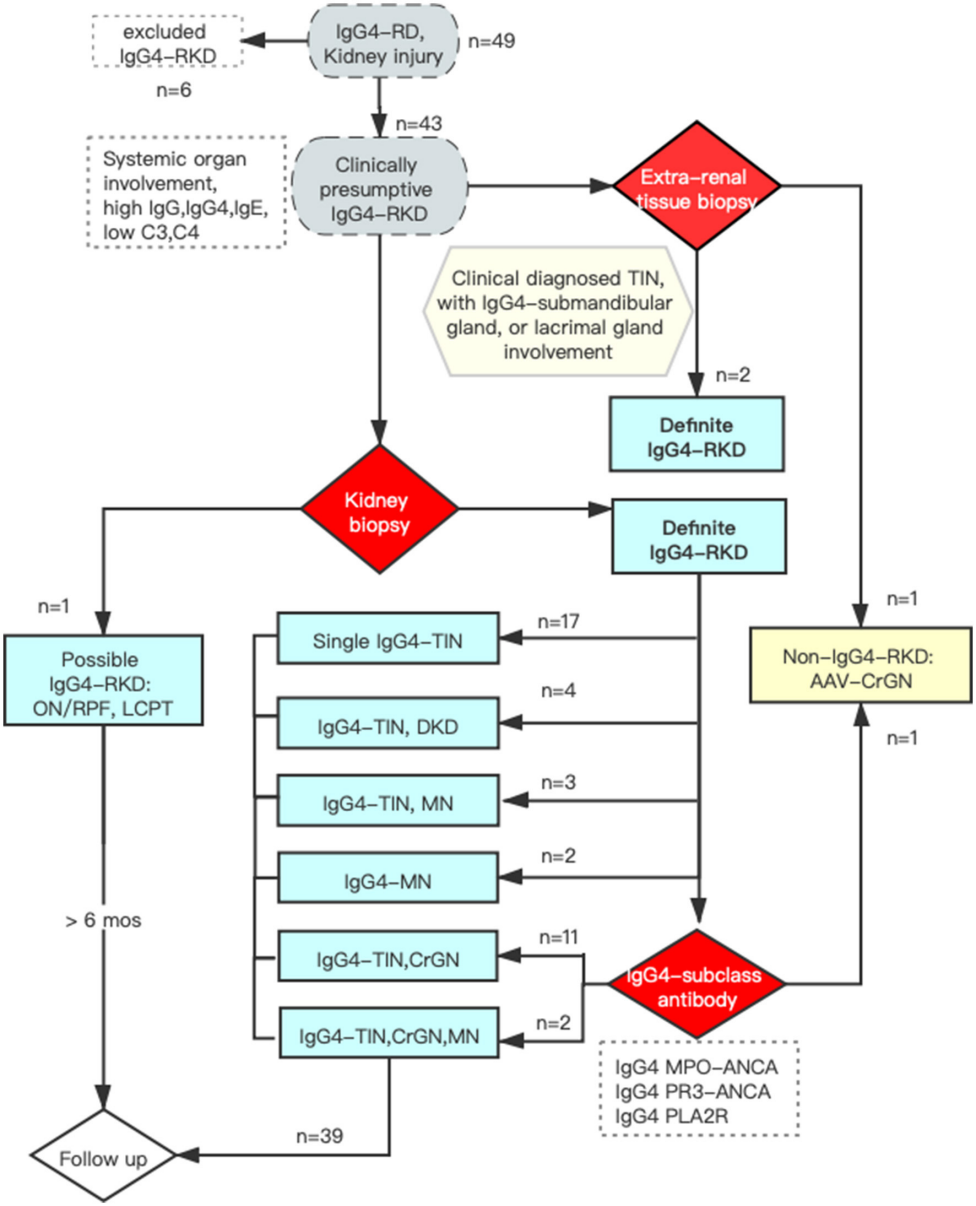
The flow chart of the study.

This study was approved by the Committee on Research Ethics of the Peking University First Hospital (2021Y042). A prospective IgG4-RKD cohort was established in 2012. IgG4-RD was diagnosed following the 2011 comprehensive diagnostic criteria (7). As shown in Figure 1, patients who were presumptively diagnosed with IgG4-RD and kidney injury were recruited initially. IgG4-RKD was confirmed using both IgG4-RKD 2011 diagnostic criteria (7) and the Raissian criteria (2). They were enrolled for further clinicopathological analysis. Those who met the above inclusion criteria but were lost to follow-up would be excluded from the study. As of December 2020, 42 patients were included (Figure 1) and followed up regularly at our specialty clinic every 3–6 months.

### Evaluation of Clinical Parameters

#### Renal Parameters

The pattern and severity of kidney injury were assessed and graded using serum creatinine (SCr) levels and the estimated glomerular filtration rate (eGFR) by the Kidney Disease Improving Global Outcome (KDIGO) clinical practice guidelines for AKI and acute kidney disease (AKD), and the KDOQI guidelines for CKD (10–12). Patients were classified into the AKD and CKD subgroups at diagnosis. A routine blood examination and liver and kidney function tests were performed. Routine urinalysis, microscopic examinations of urinary sediment, determination of the urinary albumin-to-creatinine ratio, and tubular function tests were performed in all patients. Tubular dysfunction was defined as renal glucosuria and had elevated α1-microglobulin levels.

#### lgG4-RD Parameters

Data relevant to IgG4-RD were collected, comprising a history of concurrent diseases, organ involvement, and the following inflammatory markers: erythrocyte sedimentation rate (ESR); C-reactive protein (CRP) levels; peripheral blood eosinophil count (PBEC); serum levels of IgG subclasses, IgG and IgE; and complement (C)3 and C4 levels. Eosinophilia was diagnosed if the PBEC was >0.50 × 10^9^/μL. Organ involvement was determined by physical examination and radiological findings, such as ultrasonography, CT, MRI, and positron emission tomography (PET). We used the IgG4-RD RI to evaluate systemic disease activity.

### Histopathological Examinations

Kidney biopsy specimens were collected for immunofluorescence test (IF), and light and electron microscopy analyses. Two experienced renal pathologists made the pathological diagnosis and determined the renal tubulointerstitial injury score (TiIS). The modified renal TiIS was determined by the Banff working group classification standards (13, 14), based on the severity of tubular epithelial cell injury, interstitial edema, interstitial cell infiltration, tubular atrophy, and interstitial fibrosis. The TiIS was scored from 0 to 4; the score was 0 or 1, depending on the presence or absence of tubular cell necrosis and tubulitis. The presence of active inflammation and chronic lesions was assessed by the tubulointerstitial acute injury (Ti-AI) and chronic injury (Ti-CI) scores. Additionally, we analyzed specific pathological markers including immune complex tubular basement membrane (TBM) deposition, an ectopic germinal center in the renal interstitium, storiform fibrosis, and eosinophil aggregation. The aggregation of eosinophils was categorized into mild (0–10) and severe (>10) using the total number of eosinophils in 10 visual fields at 200× magnification.

### Follow-Up and Prognosis

All patients were followed up regularly at our specialty clinic monthly for the first month and then every 3–6 months after biopsy. We performed complete blood counts, blood tests for biochemistry, ESR, CRP levels, IgG subclasses, complements, and urinalysis. The effect of immunosuppressive therapies and the changes in laboratory parameters were monitored closely. The short-term renal outcome measure was described by the decrease in the SCr level 1 month after the level peaked and classified according to the percentage decrease of the SCr level as follows: unimproved (<20% or in maintenance dialysis), partly improved (20%−40%), and significantly improved (>40%). The long-term outcomes were evaluated based on the eGFR levels during follow-up. We considered patients having a good outcome if they had an eGFR of ≥ 45 ml/min per 1.73 m^2^ at 12 months. Those who had an eGFR <45 ml/min per 1.73 m^2^ were considered to have a poor outcome. IgG4-RKD relapse was defined as an elevated SCr that exceeded 30% of the lowest value within 4 weeks. The incidence of extrarenal neoplasms during the observation period was also recorded.

### Statistical Analysis

SPSS software version 20.0 (IBM Corp., Armonk, NY, USA) was used for all statistical analyses to compare the baseline differences between patients. Continuous variables with a normal distribution, expressed as mean ± SD, were compared between groups using a *t*-test. Continuous variables with a non-normal distribution were defined as median or quartiles and were compared using the Mann–Whitney *U*-test between groups. Classified variables were expressed by the number of cases (percentage) and compared using chi-squared test, whereas ordered variables were compared using Wilcoxon rank-sum test. Correlation analysis was used to determine variables related to short-term and long-term renal outcomes. Twenty-nine risk factors including age, gender, IgG4-RD RI, ESR, CRP, total IgG and IgG subclasses, serum C3 and C4 concentration, eosinophil count or eosinophilia, pathological Ti-AI and Ti-CI scores, peak SCr at onset, and the percent of SCr decrease at 1 month after were all included as variables affecting long-term poor renal outcome and disease relapse. Least absolute shrinkage and selection operator (LASSO) regression (SAS software, version 9.4, SAS Institute Inc., Cary, NC, USA) was applied to minimize the potential collinearity and over-fitting of variables. Variables identified by LASSO regression analysis were entered into regression models. The missing values of C3, C4, IgG subclasses, CRP, and ESR were effectively complemented by a median or average data (missing three to four data per variable) for multifactorial prognostic analysis. The multivariate regression was performed to screen predictors of reversible short-term renal recovery. The logistic regression using a backward stepwise elimination was performed to determine independent risk factors for poor renal outcome and relapse. Results of the regression analyses were reported as odds ratios with 95% confidence intervals. Values of *P* < 0.05 were considered to indicate statistical significance (two-sided).

## Results

### Clinical Features

In the entire IgG4-RKD cohort, 42 patients were included in the study with a male-to-female ratio of 5:1. The mean age of the included cohort was 58.5 ± 8.7 years. The mean number of organs involved was 2.8 ± 1.4. The extra-renal organs involved were the lymph nodes (57.1%), pancreas (28.8%), salivary glands (28.8%), lungs (19.0%), hepatobiliary duct (7.1%), retroperitoneum (7.1%) showing obstructive nephropathy (ON), and large arteries (abdominal aorta, 4.8%). The mean IgG4-RD RI was 12.2 ± 3.3.

Comorbidities included hypertension (23.8%), diabetes (14.4%), hepatitis-B virus infection (4.8%), psoriasis (2.4%), skeletal fluorosis (2.4%), and pneumoconiosis (2.4%). Eight patients (19.0%) were antineutrophil cytoplasmic antibody (ANCA)-positive and met the diagnostic criteria of anti-neutrophil cytoplasmic antibody-associated vasculitis (AAV) (15, 16). Four patients had a history of carcinoma, and one each had a thymoma, carcinoma of the ureter, clear cell renal cell carcinoma, and tubular adenocarcinoma. One patient was diagnosed with extranodal marginal zone lymphoma (17). Five patients had monoclonal immunoglobulinemia, namely, IgG-κ (*n* = 3) and IgG-λ (*n* = 2).

### Features of IgG4-RKD

Laboratory findings are shown in Table 1. All patients had increased SCr levels; the mean peak SCr level was 264.5 ± 145.6 μmol/L. AKD and acute on CKD were diagnosed in 66.7% of the patients. Nine patients had high-titer autoantibodies (six with myeloperoxidase [MPO]-ANCA, two with proteinase 3 [PR3]-ANCA, and one with anti-phospholipase A2 receptor [PLA2R] antibody) and were diagnosed with crescentic glomerulonephritis (CrGN). Eight patients had nephrotic-range proteinuria. They were later pathologically diagnosed with membranous nephropathy (MN), focal segmental glomerular sclerosis, and diabetes glomerulopathy (DG). We found no cases of renal glycosuria and only one case of incomplete renal tubular acidosis. Most patients had mild-to-moderate anemia, whereas 16 patients had eosinophilia (40.0%). The median PBEC before steroid therapy was 0.4 × 10^9^/μL (quartiles: 0.1–0.74).

**Table 1:** The clinical and pathological features of IgG4-related kidney disease.

	**AKD**		**CKD**	***P*** **-value**
	**ANCA** ***N =*** **8**	w/o ANCA** ***N =*** **19**	**CKD** ***N =*** **12**	
Duration of follow-up (month)	29.2 ± 20.1	40.8 ± 35.0	61.7 ± 32.0	0.118
Age (yr)	59.3 ± 9.6	57.5 ± 9.0	58.4 ± 8.8	0.89
Gender M/F	5/3	16/3	11/1	NS
**IgG4-RD parameters**				
ESR (mm/1 h)	104.3 ± 31.1	86.4 ± 46.5	55.0 ± 31.9	0.030
CRP (mg/L)	52.5 ± 35.8 (10.9–104)	18.3 ± 25.9 (1.77–24.5)	7.7 ± 16.6 (0.23–57.2)	0.002
Responder index	10.4 ± 3.3	13.7 ± 3.5	10.8 ± 2.3	0.013
Organs involved	1.5 ± 0.5	3.3 ± 1.5	2.6 ± 0.8	0.040
IgG (g/L)	26.4 ± 8.2	48.7 ± 42.5	32.3 ± 33.9	0.251
IgG1 (g/L)	14.2 ± 4.8	17.8 ± 14.9	11.4 ± 4.2	0.409
IgG2 (g/L)	6.4 ± 2.2	6.9 ± 6.5	4.5 ± 2.0	0.546
IgG3 (g/L)	0.9 ± 0.8	1.2 ± 1.1	1.2 ± 1.0	0.742
IgG4 (g/L)	5.0 ± 3.0	22.9 ± 26.4	23.2 ± 29.1	0.197
IgG4/IgG (%)	18.3 ± 10.3	35.6 ± 22.8	42.3 ± 31.1	0.099
C3 (g/L) Low C3	0.79 ± 0.17 1/7 (14.3%)	0.55 ± 0.24 12/17 (70.6%)	0.64 ± 0.27 7/13 (53.8%)	0.104
C4 (g/L) Low C4	0.20 ± 0.10 0	0.09 ± 0.09 10/17 (58.8%)	0.13 ± 0.11 10/17 (58.8%)	0.038
**IgG4-RKD parameters**				
Kidney length (cm)	10.7 ± 0.6	12.7 ± 1.7	12.0 ± 1.2	0.650
PBEC (x 10^9^)High PBEC before steroids	0.9 ± 1.1 3/7 (42.9%)	1.1 ± 2.0 9/16 (56.3%)	0.4 ± 0.3 4/13 (30.8%)	0.384
ACR (mg/g)	149.7 ± 186.4	638.5 ± 1329.3	392.7 ± 582.1	0.545
α1-microglobulin (mg/L)	209 ± 297	106.0 ± 53.5	94.0 ± 112.4	0.916
SCr max (μmol/L)	374.8 ± 190.6	314.1 ± 135.5	169.8 ± 60.6	0.003
SCr at 1 month (μmol/L)	253.4 ± 101.0	201.1 ± 102.6	134.0 ± 42.3	0.016
**Disease response**				
Scr decrease 1st month (%)	0.19 ± 0.23	0.31 ± 0.14	0.16 ± 0.10	0.021
max during follow-up (%)	0.38 ± 0.29	0.52 ± 0.16	0.30 ± 0.13	0.015
**IgG4-RKD histological index**				
**Pathological type**				
TINY/N	8/0	19/0	10/2	N.D.
Glomerulopathy Y/N	8/0	10/9	7/5	N.D.
MN	0	5 (2, PLA2R+)	3	N.D.
DG	0	2	1	N.D
CrGN	8	3	2	N.D.
FSGS	0	0	1	/
Ti-AI	4.6 ± 1.1	4.5 ± 1.1	3.8 ± 1.8	0.26
Ti-CI	4.6 ± 1.8	6.1 ± 2.0	5.7 ± 2.7	0.319
Eosinophil aggregationY/N	3/5, 37.5%	13/6, 68.4%*	10/2, 66.7%	0.064
TBM deposit Y/N	2/6, 25.0%	13/6, 68.4%	3/9, 25.0%	0.190
Storiform fibrosisY/N	3/5, 37.5%	10/9, 52.6%	3/9, 25.0%	0.576

* *Significance existed between AKD subgroups*.

** *The patient with obstructive nephropathy by retroperitoneal fibrosis and LCPT was not included in the comparison*.

*ND, no data*.

All patients showed intumescent kidneys. Scar formation was found in two patients. Five patients showed multiple low-density lesions of the kidneys in CT scans. The SUV_*max*_ values of high-uptake kidney lesions in the PET-CT examination (*n* = 6) ranged from 3.7 to 11.1.

### Characteristics of Inflammatory Immune Indices of IgG4-RKD

The median CRP level was 6.45 mg/L (2.84–24.5; reference range: 0–8 mg/L), and the mean ESR was 83.7 ± 40.3 mm/h. Elevated serum IgG levels were observed in 88.1% of all the patients; IgM and IgE levels were elevated in 2.4 and 75.0% of the patients, respectively. The prevalence of hypocomplementemia was 69.0% for C3 and 50.0% for C4. Increased IgG1, IgG2, IgG3, and IgG4 levels were found in 86.8, 28.9, 52.6, and 94.7% of the patients, respectively (IgG subclasses were not tested in two patients). We found that the MPO–ANCA was IgG1 plus IgG4 in five patients, IgG3 plus IgG4 in one patient, and IgG4 in one patient. PR3-ANCA was IgG4 in one patient, and PLA2R antibody was IgG3 plus IgG4 in another patient.

All IgG4-RKD patients with overlapped AAV were grouped in the AKD subgroup having extremely high ESR and CRP levels compared with the non-AAV patients (Table 1). However, non-AAV AKD patients had a higher IgG4-RD RI, PBEC, organ involvement, and prevalence of low C3 and accelerated ESR than patients in the CKD subgroup. Based on the comparison, IgG4-RKD patients are noted to have eosinophilia with a higher prevalence of AKD and interstitial eosinophil aggregation and significantly elevated IgG4-RD RI, IgG, and IgG4 levels.

### Pathological Features of IgG4-RKD

#### General Pathological Features

**Figure 2:**
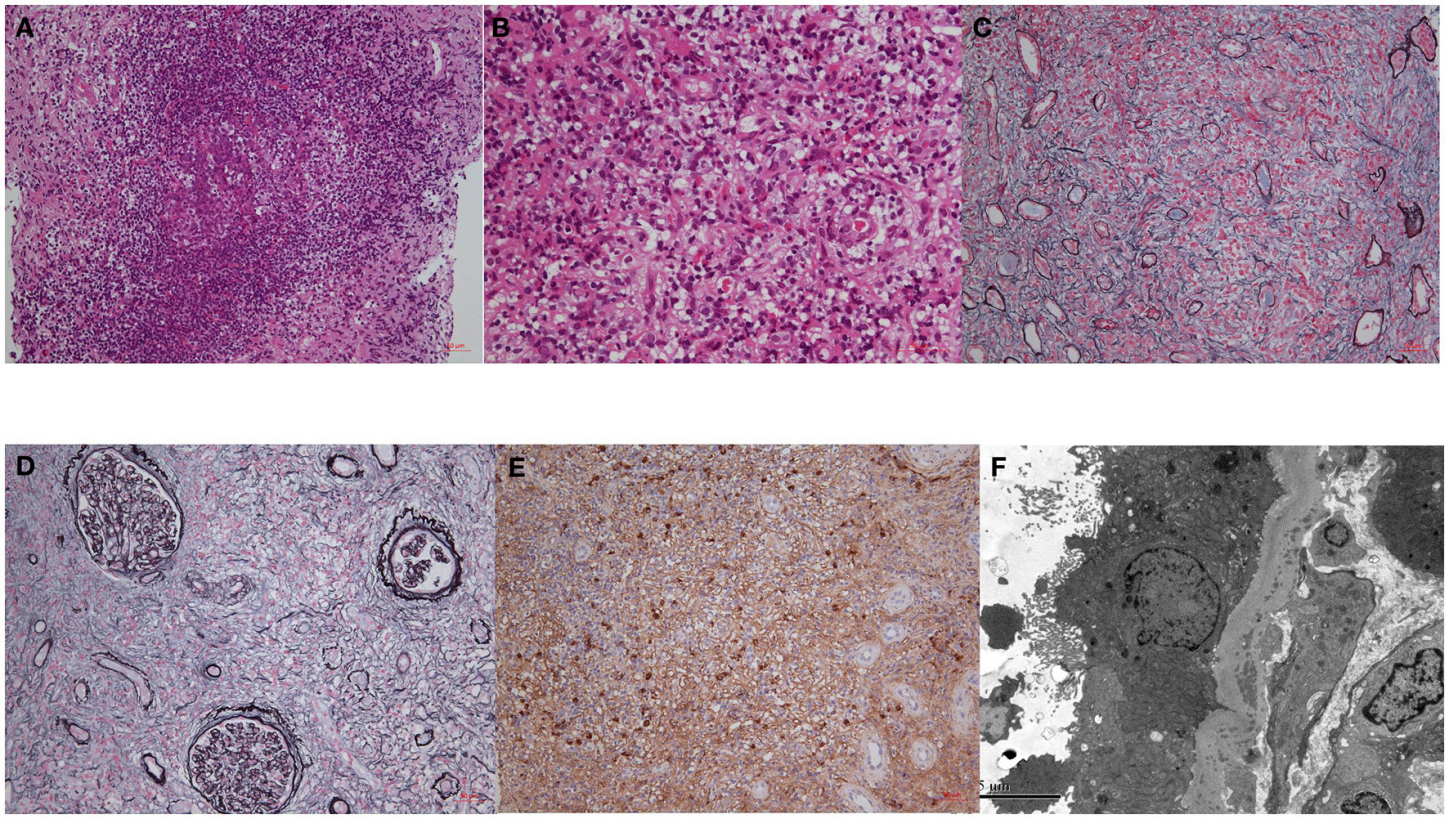
The pathological findings of IgG4-RKD. **(A)** An ectopic lymphoid tissue; **(B)** eosinophils infiltration; **(C,D)** storiform fibrosis; **(E)** IgG4-possitive staining plasma cells. **(F)** TBM deposits by EM.

A kidney biopsy was conducted in 40 patients. The renal pathology showed typical IgG4-tubulointerstitial nephritis (IgG4-TIN) in most (90.0%) patients (Figure 2); 17 patients (42.5%) were diagnosed with single IgG4-TIN, two with single IgG4-MN, and 19 (47.5%) with IgG4-TIN and concurrent glomerulopathy (Supplementary Tables 1, 2). Manifestations of glomerular involvement mainly included MN, CrGN or glomerular crescent formation, and diabetic glomerulopathy. Other types had focal segmental glomerular sclerosis, endocapillary proliferative glomerulonephritis, Light-chain proximal tubulopathy (LCPT) and IgA nephropathy. Glomerular crescent was common, and 61.5% of these were ANCA-positive. There were eight MN cases; five occurred before IgG4-RD diagnosis, one occurred after IgG4-RD diagnosis, and the other two were detected along with IgG4-RD. The densely distributed interstitial infiltrates consisted of lymphocytes, plasma cells, monocytes/macrophages, and eosinophils. Despite a 40.0% incidence of eosinophilia, we found 77.5% of the patients presenting with interstitial eosinophil infiltration and 83.9% of them (65.0% of the total) had severe aggregation. Repeated renal biopsies revealed elimination of diffusely infiltrated eosinophils, along with a decline in the SCr levels of 17.2–36.9% after steroid administration in two patients (#1 and #2; Table 2).

**Table 2:** Pathological findings of patients with repeat biopsy.

	**Time interval**	**Kidney pathology**	**ANCA**	**Anti-PLA2R**	**IF**	**TBM deposit**	**Storiform fibrosis**	**Eosinophil infiltration**	**AI**	**CI**	**Treatment**	**Outcome**
No. 1	4 wks	ATIN	/	/	G: negative TI: G1-G2-G3-G4++	0	Y	Y	4	5	Pred	Scr 198
		CTIN	/	/	G: negative TI: G1-G2-G3-G4+	0	Y	N	0	8	Pred+CTX	Scr 125
No. 2	6 wks	CrGN, ATIN	MPO	neg	G: negative	0	Y, locally	Y	5	2	MP bolus,	Scr 157-130
		CrGN, CTIN	Neg	neg	G: negative	0	Y	N	3	7	Rituximab	Scr 149
No. 3	3 yrs	MN, ATIN	/	/	G: negative	Y	Y, locally	Y, mild	4	4	Pred	Scr 138-119, 165
		MN, ATIN/CTIN	neg	neg	G: G1+G2-G3-G4+ C3++ PLA2R- TI: G1++G2+G3+G4++	Y	Y	Y	5	7	Pred+CTX	Scr 281
No. 4	2 yrs	MN	/	neg	G: G1+++G2++G3++G4+ ++C3+	Y	N	N	1	2	Pred+CsA	/
		MN,TIN, CrGN	neg	neg	G: G1++G2+G3+G4++ C3++ PLA2R- TBM: G+ C3+ G4+ TI: G1++G2+G3+G4++	Y	Y, locally	Y, locally	5	6	MP bolus	Scr 552

*ANCA, antineutrophil cytoplasmic antibody; MN, membranous nephropathy; CrGN, crescent glomerulonephritis; TIN, tubulointerstitial nephropathy; Pred, prednisone; CTX, cyclophosphamide; CsA, cyclosporin A; MP, methyprednisolone; AI, tubulointerstitial acute injury; CI, tubulointerstitial chronic injury*.

The IF staining features of glomeruli are shown in Supplementary Table 1. Among patients with crescent formation, IgG1, IgG2, and IgG3 were positive in 37.5% of the patients, and IgG4 was positive in 45.0% of the patients. Examination of IF combined with electron microscopy revealed restricted λ-light chain expression in two patients [one in the glomeruli by IF, and the other in the interstitium (17) by EM] and κ-light chain in one. Renal pathology revealed that 19 patients (47.5%) had TBM deposits and 52.6% of them had glomerular lesions. Seventeen patients (42.5%) showed storiform fibrosis during routine light microscopy, whereas six (15.0%) patients showed renal interstitial ectopic lymphoid tissue.

#### Findings of Kidney Rebiopsy

Two patients (#1 and #2) received rebiopsy 4–6 weeks after a strengthened immunosuppressive treatment. The Ti-AI score was extremely lowered. Another two patients (#3 and #4) underwent a repeated biopsy after an ~2-year interval. The initial pathological finding was of MN with TBM deposits. Proteinuria and AKD relapsed several months after steroid withdrawal. The Ti-AI and Ti-CI scores were both elevated, together with interstitial eosinophil infiltration (Table 2).

### Treatments and Disease Response

All patients were initially prescribed prednisone (0.5–1.0 mg/kg/day) for 4–8 weeks, which was regularly tapered by 5 mg every 2 weeks. Six patients (14.3%) with CrGN received methylprednisolone–pulse therapy combined with plasmapheresis. Nine (21.4%) patients had single steroid therapy. Twenty-one patients were given cyclophosphamide (50.0%), mycophenolate mofetil (4.8%), or other conventional immunosuppressive agents (azathioprine or cyclosporine, 4.8%) combined with steroids. Eight patients (19.0%) received a rituximab regimen. For all, prednisone administration was maintained at 5–10 mg/day.

The patients were followed up for 45.4 ± 33.8 months. In 1 month of immunosuppressive therapy, the reversible recovery part of renal function was as follows: (1) unimproved in 14 patients (35.0%), (2) partly improved in 17 patients (40.5%), and (3) significantly improved in nine patients (22.5%). All patients had CKD at 12 months, with CKD stages 3–5 accounting for 50.0, 10.0, and 7.5%, respectively. All patients had remission of hypocomplementemia. Unfortunately, elevated IgG4 level (median at 1.6, quartile: 0.57, 6.0 g/L) remained, involving 47.8% of patients during maintenance immunosuppressive therapy, and the percent of uncontrolled non-IgG4 subclass was found to be 16.7–22.2%. Thirteen patients (31.0%) presented with an IgG4-RKD relapse. Recurrent eosinophilia was present in another four patients (9.5%). Before relapse, six patients (46%) received a single steroid; combination strategies included cyclosporin A, cyclophosphamide, mycophenolate mofetil, azathioprine, and the R-CHOP regimen. Four patients developed new neoplasms (lung cancer, esophageal cancer, and diffuse large B-cell lymphoma). The prevalence of malignancies in the current cohort was 21.4%.

### Correlation Analysis of the Clinicopathological Parameters for Predicting IgG4-RKD Activity and Renal Outcomes

#### Factors Affecting Renal Outcomes

Correlation analysis showed that the clinical parameters including IgG4-RD RI, total IgG, IgG subclass levels, PBEC, and the IgG4/IgG ratio were closely correlated. IgG4-RD RI was also correlated to serum C3 (*R* = −0.509, *P* = 0.001), C4 (*R* = −0.314, *P* = 0.049) levels, and PBEC (*R* = 0.377, *P* = 0.024), factors that were not included in RI scores. The systemic inflammatory indicator ESR was found to correlate with IgG1 (*R* = 0.350, *P* = 0.043), and CRP correlated with serum C3 level (*R* = 0.544, *P* = 0.001). Pathological Ti-AI had an inverse relationship with serum IgG4 (*R* = −0.399, *P* = 0.014). Ti-CI correlated with serum C3 (*R* = −0.448, *P* = 0.006) and IgG3 (*R* = 0.374, *P* = 0.027). Renal eosinophil infiltration existed when there was eosinophilia (*R* = 0.447, *P* = 0.007) and TBM deposit (*R* = 0.95, *P* = 0.00). Correlation analysis further disclosed that IgG4-RD RI (*R* = 0.422, *P* = 0.007), organs involved (*R* = 0.452, *P* = 0.003), and C3 (*R* = −0.487, *R* = 0.002) were correlated with the percentage decrease of SCr at 1 month (Table 3). However, multivariate regression analysis failed to identify any clinicopathological parameters that could predict good short-term renal outcome and IgG4-RKD relapse. Comparison displayed in Table 4 revealed that patients with an eGFR ≥ 45 ml/min per 1.73 m^2^ at 12 months had a significantly higher PBEC and serum IgG4 level, whereas the peak Scr at disease onset was relatively lower.

**Table 3:** The affecting factors to percentage decrease of Scr at 1 month.

**Variable**	**Unimproved**	**Partly improved**	**Significantly improved**	***P***
	***N =*** **14**	***N =*** **17**	***N =*** **9**	
Age, mean (SD)	61.857 (9.418)	55.353 (7.638)	58.667 (7.498)	0.125
Gender/male, n(%)	12 (85.714)	15 (88.235)	6 (66.667)	0.359
IgG4-RD RI, mean (SD)	10.071 (2.404)	12.353 (2.195)	14.778 (4.315)	0.003
Organs involved, median [IQR]	2.0 [1.0, 3.0]	3.0 [2.0, 4.0]	4.0 [2.0, 5.0]	0.02
Eosinophilia, n(%)	7 (50.000)	5 (31.250)	4 (66.667)	0.286
SCr decrease %, mean (SD)	0.073 (0.104)	0.270 (0.060)	0.449 (0.039)	<0.001
SCr max, median [IQR]	160.0 [136.8, 372.0]	243.0 [191.0, 322.0]	352.0 [276.0, 514.9]	0.061
CRP, median [IQR]	3.85 [2.6, 10.88]	9.99 [2.84, 38.8]	6.45 [4.95, 24.0]	0.78
ESR, median [IQR]	71.0 [52.0, 116.0]	81.47 [64.0, 92.0]	100.0 [86.0, 126.0]	0.546
PBEC, median [IQR]	0.4 [0.12, 0.6]	0.15 [0.1, 0.5]	0.55 [0.2, 3.0]	0.168
IgG1, median [IQR]	12.4 [10.24, 13.1]	12.6 [12.1, 14.6]	14.4 [9.69, 24.0]	0.824
IgG2, median [IQR]	4.59 [2.74, 5.79]	5.62 [4.63, 7.09]	5.3 [4.79, 6.04]	0.398
IgG3, median [IQR]	0.754 [0.338, 1.09]	1.41 [0.385, 1.49]	0.635 [0.3, 2.25]	0.383
IgG4, median [IQR]	8.76 [1.86, 13.0]	8.35 [3.49, 29.6]	7.62 [4.0, 49.2]	0.446
C3, median [IQR]	0.616 [0.51, 0.94]	0.573 [0.44, 0.842]	0.397 [0.313, 0.514]	0.033
C4, median [IQR]	0.14 [0.052, 0.262]	0.12 [0.07, 0.228]	0.11 [0.021, 0.129]	0.257
Ti-AI, median [IQR]	4.0 [4.0, 6.0]	4.0 [4.0, 5.0]	5.0 [4.0, 5.0]	0.842
Ti-CI, median [IQR]	6.0 [4.0, 8.0]	6.0 [4.0, 7.0]	6.0 [5.0, 7.0]	0.893
With concurrent GN, n(%)	11 (78.571)	6 (37.500)	5 (55.556)	0.077
Eon infiltration, n(%)	7 (50.000)	9 (56.250)	4 (44.444)	0.845
Storiform fibrosis, n(%)	11 (78.571)	8 (50.000)	4 (44.444)	0.17

**Table 4:** The comparison between patients with good or poor long-term prognosis.

**Factors**	**eGFR≥45 (***n =*** 20)**	**eGFR <45 (***n =*** 20)**	***p***
Gender/male, n(%)	18 (90.000)	15 (75.000)	0.212
Age, mean (SD)	56.500 (7.487)	60.250 (9.497)	0.184
Scr max, median [IQR]	178.3 [136.8, 243.0]	352.0 [282.9, 417.0]	<0.001
ΔScr at 1 month, mean (SD)	0.227 (0.167)	0.256 (0.151)	0.586
Eosinophilia, n(%)	12 (70.588)	4 (21.053)	0.003
IgG4-RKD relapse, n(%)	5 (25.000)	8 (40.000)	0.311
A1MG, mean (SD)	97.056 (89.331)	307.123 (569.644)	0.172
CRP, mean (SD)	22.543 (34.710)	21.892 (24.053)	0.949
ESR, mean (SD)	72.530 (45.560)	90.444 (34.773)	0.201
RI, mean (SD)	12.750 (2.791)	11.450 (3.748)	0.233
Organs involved, median [IQR]	3.0 [2.0, 3.0]	2.0 [1.0, 4.0]	0.445
Total IgG, mean (SD)	50.792 (39.172)	35.479 (26.989)	0.193
IgG1, mean (SD)	14.827 (13.329)	16.193 (8.525)	0.724
IgG2, mean (SD)	5.064 (2.237)	7.353 (6.345)	0.17
IgG3, mean (SD)	1.003 (0.790)	1.294 (1.134)	0.391
IgG4, mean (SD)	29.898 (29.239)	10.262 (13.918)	0.019
C3, mean (SD)	0.642 (0.252)	0.593 (0.256)	0.57
C4, mean (SD)	0.150 (0.107)	0.166 (0.171)	0.74
With concurrent GN, n(%)	12 (60.000)	10 (52.632)	0.643
Eon infiltration, n(%)	12 (60.000)	8 (42.105)	0.264
Germalcentre, n(%)	3 (15.000)	3 (15.789)	0.946
Storiform fibrosis, n(%)	5 (25.000)	11 (57.895)	0.037
TBM, n(%)	11 (55.000)	8 (42.105)	0.421
CI, mean (SD)	5.500 (2.037)	5.789 (2.330)	0.689
AI, mean (SD)	4.150 (1.558)	4.421 (1.042)	0.54

Ten out of the 29 variables of most importance were identified by LASSO regression analysis. They were included in the multivariate logistic regression, including age, serum IgG subclasses, C3, C4, ESR, CRP, and PBEC. Independent predictors for short-term reversible renal recovery, IgG4-RKD relapse, and long-term poor outcome would be identified. By multivariate logistic regression, a lower serum IgG4 (O*R* = 0.671, *P* = 0.010), higher IgG1 (OR = 1.396, *P* = 0.049), IgG3 (O*R* = 19.154, *P* = 0.039), and ESR (O*R* = 1.042, *P* = 0.032) were found to be independent factors of poor long-term outcome (Tables 4, 5).

**Table 5:** The adjusted odds ratio of variables for poor long-term outcome evaluated by eGFR (<45 ml/min per 1.73 m^2^) at 12 months.

**Input variable**	**Adjusted OR (95% CI)**	***P***
Age		0.086
C3		0.377
C4		0.121
IgG1	1.396 (1.001–1.947)	0.049
IgG2		0.961
IgG3	19.154 (1.168–314.216)	0.039
IgG4	0.671 (0.496–0.907)	0.010
ESR	1.042 (1.003–1.082)	0.032
CRP		0.057
PBEC		0.174

## Discussion

This study includes the clinical data of the largest cohort of patients with biopsy-proven IgG4-RKD at the Peking University Institute of Nephrology. Our study showed that the onset of IgG4-RKD was insidious and that IgG4-TIN was the typical renal manifestation of IgG4-RKD, which was congruent with the findings of previous reports (2). Our results suggested that most (83.3%) of the patients with CKD who underwent kidney biopsy had active tubulointerstitial inflammation and achieved significant restoration in the renal function after receiving immunosuppressive therapies (18). This finding also suggests that the creatinine-based glomerular function assessment method or the tubular dysfunction markers are not sensitive in detecting early interstitial inflammation, although some weak correlations were observed between IgG subclass, hypocomplementemia, and Ti-IS scores. More specific and sensitive indicators are yet to be found. Moreover, we reported a high prevalence and diversity of glomerular diseases in patients with IgG4-RD, especially CrGN or crescent formation, which is critically important and significantly increases the difficulty of clinical evaluation of IgG4-RKD. In other words, the deterioration or recurrence of IgG4-RKD may result from aggravated interstitial inflammation or the occurrence of severe glomerular involvement. For these reasons, biopsy remains the cornerstone of diagnosis to evaluate the renal pathology precisely and minimize missed diagnoses of active inflammation (19). Saeki et al. (20) reported that the revised version of the diagnostic criteria for IgG4-RKD (2020), which adds items pertaining to the extra-renal organ(s) (including “bilateral lacrimal, submandibular, or parotid swelling and imaging findings compatible with type 1 autoimmune pancreatitis or retroperitoneal fibrosis”), helps to improve both the true IgG4-RKD diagnostic sensitivity and specificity. Nevertheless, further investigation of non-invasive indicators reflecting active intrarenal inflammation is still needed (21).

Eosinophilia is a common manifestation of IgG4-RD and is believed to be a sign of an enhanced immune response (19, 22). Renal eosinophilic infiltration is also frequently observed pathologically in IgG4-RKD. It is reported in up to 30.8% of the patients, significantly higher than 9.5% in non-IgG4-RKD (23). Recently, research has shown that eosinophils are involved in the progression of CKD. Rats with eosinophil peroxidase deficiency were reported to have mild renal interstitial fibrosis (24). Zachary et al. (25) observed that elevated PBEC at baseline was a potential predictor of IgG4-RD relapse. Zhang et al. (26) showed that patients with IgG4-RD and eosinophilia had more organ involvement, especially lymph nodes, lacrimal gland, and submandibular gland. Mohapatra et al. (27) reported that eosinophilia with increasing serum IgG4 added diagnostic value at serum IgG4 levels of ≥ two times the upper limit of normal. Takanashi et al. (28) conducted proteomic analysis in IgG4-RD and showed that eotaxin-3 was a new biomarker of disease activity. Our results showed that eosinophilia represented a more active IgG4-RKD and indicated reversible treatment response. Interstitial eosinophilic aggregation was more significant in IgG4-TIN and required immunosuppressive therapy. However, a 35% missed diagnosis rate was noted because patients with obvious eosinophilic infiltration had a normal PBEC. A kidney biopsy could correct a missed diagnosis.

The number of case reports and cohort studies regarding IgG4-RD overlapping with ANCA-positive CrGN, systemic lupus erythematosus (SLE), Sjogren's syndrome, and IgG4-associated MN and TMA have been rising (19, 26, 29–33). Some researchers believe that MN is a special type of IgG4-RKD, regardless of whether PLA2R is negative (34). A study reported 65 IgG4-RD patients with urinary involvement, 7.7% of whom had MN (3). A cohort with type-1 autoimmune pancreatitis and kidney involvement reportedly comprised 26.7% of patients with MN (4). In another IgG4-RD cohort with retroperitoneal involvement, 27.4% of the patients presented with IgG4-TIN and MN (35). The difference is that the present study reported more types of glomerulopathy, especially CrGN induced by IgG4 subtype ANCA and anti-PLA2R. If accompanied by an AAV, patients usually showed different characteristics from that of typical IgG4-TIN, which has an acute onset of AKI with elevated CRP and a rarely detected hypocomplementemia. Martín-Nares et al. (36) also emphasized that a significant number of patients with IgG4-RD were ANCA-positive and more frequently had lymph node and kidney involvement, high IgG1 levels, and high ESR. Wang et al. found similar presentations in four patients with the concurrent crescent formation in AAV and IgG4-TIN (37). Our current study showed that the pathogenicity of IgG4 subtypes specific to ANCA and PLA2R should be classified as IgG4-RKD. Furthermore, we disclosed other patterns of glomerulopathy, such as non-ANCA crescents formation, restricted light chain expression, and concurrent diabetes glomerulopathy, which were also involved in kidney injury.

Even though some IgG4-recognized target antigens have been found (38–41), IgG4 does not bind to activating Fc receptors as a driver of pathogenesis. An increase in the IgG subtypes other than the IgG4 molecules and hypocomplementemia is also frequent in IgG4-RD, but their roles remained unclear. Our study showed that elevated IgG1, IgG2, and IgG3 levels occurred in 86.8, 28.9, 52.6% of the patients, respectively. Low C3 and C4 levels accounted for 69 and 50% of all the patients, respectively. Previous studies with biopsy-proven IgG4-RKD showed that C3 levels were negatively correlated with the renal deposition of C1q, storiform fibrosis, interstitial fibrosis, the number of IgG4-positive plasma cells (42), and deposition of non-IgG4 immunoglobulins in the TBM (43). This was similar to our results that high C3 and IgG3 levels were significantly correlated to the severity of the tubulointerstitial chronic injury. The literature also reports that IgG3 antibodies, such as IgG3-ANCA, can lead to robust complement activation. IgG1 and IgG4 anti-PR3 ANCA can induce degranulation and adhesion of granulocytes (41). Disease severity (44) is associated with an increase in the diversity of autoantibodies in IgG4-RD.

Patients show different characteristics of IgG4-RKD and progression, which correspond to the acceleration or remission of the immune response. Takanashi et al. (28) reported that IgG4-lymphadenopathy represented a phenotype of severe activity and poor prognosis. Miyanaga et al. (45) reported tertiary lymphoid tissue, a potential marker of relapse and drug resistance of IgG4-RKD, in an early stage of IgG4-TIN, related to poor renal prognosis. Chibbar et al. (46) reported recurrent IgG4-related TIN concurrent with chronic active antibody-mediated rejection, despite maintenance immunosuppression. These observations indicate that B- and T-cells are vital to disease pathogenesis. T-follicular helper cells, residing in germinal centers, are elevated in the peripheral blood and affected tissues, promoting B-cell differentiation, expansion, and germinal-center formation. Regulatory T and Th2 cells phenotype stimulate cytokine production (which promotes fibrosis) and an Ig class switches to IgG4 (19, 34). Therefore, conventional immunosuppressive and B-cell-depletion therapies can cause a rapid decline in the production of IgG subclasses and reduce inflammation and fibrosis. However, the risk of disease relapse and tumorigenesis remains a dilemma in clinical treatment (47). In IgG4-RD, serum IgG4 levels decline after B cells depletion but do not normalize, probably because of the long-lived plasma cells that continue to produce it, which result in persistently elevated immunoglobulin. Recently, it is reported that circulating plasmablasts correlate well with disease activity and the number of the organs involved, making them a useful biomarker of IgG4-RD differentiating from mimickers and during disease relapse. The limitation of this study was that we did not measure baseline CD19^low^CD20^neg^CD27^+^CD38^hi^plasmablasts at diagnosis.

In conclusion, this study indicates that IgG4-RKD patients have complex and diverse clinicopathological manifestations. In the absence of sensitive and specific predictors, pathological kidney findings are still the most reliable basis for diagnosing, assessing, and treating IgG4-RKD. A higher serum IgG1, IgG3, and ESR were independent factors for the poor long-term renal outcome; however elevated IgG4 predicted a good renal prognosis. An appropriate and timely immunosuppressive therapy is helpful in improving renal function restoration.

## Data Availability Statement

The original contributions presented in the study are included in the article/Supplementary Material, further inquiries can be directed to the corresponding author/s.

## Ethics Statement

The studies involving human participants were reviewed and approved by the Ethics Committee of Peking University First Hospital (2021Y042). The patients/participants provided their written informed consent to participate in this study.

## Author Contributions

TS and LY participated in research design and data analysis. TS, LY, HW, and SW participated in the performance of the study. TS drafted the manuscript. All authors contributed to the article and approved the final manuscript for submission.

## Funding

This research was supported by the National Science and Technology Major Projects for major new drugs innovation and development (Grant No. 2017ZX09304028 to TS), National Natural Science Foundation of China (Grant Nos. 91742205 and 81625004 to LY), and the Chinese Academy of Medical Sciences Innovation Fund for Medical Sciences (Grant No. 2019-I2M-5-046).

## Conflict of Interest

The authors declare that the research was conducted in the absence of any commercial or financial relationships that could be construed as a potential conflict of interest.

## Publisher's Note

All claims expressed in this article are solely those of the authors and do not necessarily represent those of their affiliated organizations, or those of the publisher, the editors and the reviewers. Any product that may be evaluated in this article, or claim that may be made by its manufacturer, is not guaranteed or endorsed by the publisher.

## Abbreviations

IgG4-RKD: Immunoglobulin-4–related kidney disease
ANCA: antineutrophil cytoplasmic antibody
MPO: myeloperoxidase
PR_3_: proteinase 3
PLA_2_R: phospholipase A_2_ receptor
AAV: Anti-neutrophil cytoplasmic antibody-associated vasculitis
TBM: tubular basement membrane
AKI: acute kidney injury
AKD: acute kidney disease
CKD: chronic kidney disease
CrGN: crescentic glomerulonephritis
MN: membranous nephropathy
CRP: C-reactive protein
ESR: erythrocyte sedimentation rate
TI-AI: tubulointerstitial acute injury score
RI: responder index.
