# Supplementary material for: Clinicopathological Patterns and Predictors of the Functional Restoration of Immunoglobulin G4-Related Kidney Disease: A Chinese Single-Center Cohort Study
Source: Front Med (Lausanne). 2021 Oct 6;8:736098. doi: 10.3389/fmed.2021.736098 (PMC8526789; doi:10.3389/fmed.2021.736098)
Supplement: Supplementary file 1 [file Table_1.DOCX]

**Table S1 Pathological findings of glomerulopathy patterns in IgG4-RKD**

|  | **TIN types** | **Glomerular types** | **Auto-antibodies** | **IF staining in Glomeruli:**  **IgG,C_3_,C1q,IgG_1_,IgG_2_,IgG_3_,IgG_4_,PLA_2_R,k,λ** | **TBM**  **deposit** |
| --- | --- | --- | --- | --- | --- |
| ***IgG4-relating glomerulopathy*** | | |  |  |  |
| Case 1 | IgG4-TIN | MN | none | IgG++C3++, IgG1+IgG2++IgG4++ | Y |
| Case 2 | IgG4-TIN | MN | none | IgG+++C3+, IgG1++IgG4+, PLA_2_R+++ | Y |
| Case 3 | IgG4-TIN | MN | none | IgG+C3++, IgG1+IgG4+, PLA2R- | Y |
| Case 4 | IgG4-TIN | MN, CrGN | anti-PLA_2_R (IgG_3_,IgG_4_) | IgG+++C3+++C1q++, IgG1++IgG2+IgG3++IgG4++, PLA_2_R++ | Y |
| Case 5 | IgG4-TIN | MN, CrGN | none | IgG+++C3+++IgG1++IgG2+IgG3+IgG4+++ | Y |
| Case 6 | / | MN | none | IgG++++C3+++IgG1+++IgG2+G4++, PLA2R- | N |
| Case 7 | / | MN | none | IgG+++C3+IgG1+++IgG2+IgG4+++, PLA2R+ | N |
| Case 8 | IgG4-TIN | Crescents 29% | none | IgG++C3+C1q+IgG1+IgG2+IgG3+IgG4+ | N |
| Case 9 | IgG4-TIN | CrGN, IgAN | none | C3+++IgG4- | N |
| Case 10 | IgG4-TIN | Cresencts, 44% | none | IgG+C3+++ | N |
| Case 11 | IgG4-TIN | Crescents, 20% | MPO-ANCA  (IgG_1_,IgG_4_) | All negative | N |
| Case 12 | IgG4-TIN | Crescents, 15% | PR3-ANCA (IgG_4_) | C3+ | N |
| Case 13 | IgG4-TIN | CrGN | MPO-ANCA (IgG_1_,IgG_4_) | C3++, IgG2+IgG3+IgG4++ | N |
| Case 14 | IgG4-TIN | Crescents, 23% | MPO-ANCA (IgG_4_) | IgG+IgG2+ | Y |
| Case 15 | IgG4-TIN | CrGN | MPO-ANCA (IgG_1_,IgG_4_) | C3+ | N |
| Case 16 | IgG4-TIN | CrGN | MPO-ANCA (IgG_3_,IgG_4_) | All negative | N |
| Case 17 | IgG4-TIN | CrGN | PR3-ANCA (IgG_3_,IgG_4_) | C3++IgG4+ | Y |
| Case 18 | IgG4-TIN | CrGN | MPO-ANCA (IgG_3_,IgG_4_) | IgG++C3+++, IgG1++IgG2+IgG3++IgG4++ | N |
| ***Undetermined IgG4-relating glomerulopathy*** | | | |  |  |
| Case 19 | IgG4-TIN | DG, thick TBM | DKD | All negative | N |
| Case 20 | IgG4-TIN | DG | DKD | IgG+C3+++, λ+++ | Y |
| Case 21 | IgG4-TIN | FSGS | none | C3++ | Y |
| Case 22 | IgG4-TIN | DG, thick TBM | DKD | IgG+++C3+++C1q++IgG2+IgG4+ | N |
| Case 23 | IgG4-TIN | EGPA | Unknown | All negative | N |
| Case 24 | / | LPTD^ | MGRS | All negative | N |

Note: AAV, ANCA-positive vasculitis; MN, membranous nephropathy; CrGN, crescent glomerulonephritis; EGPA, Endocapillary proliferative glomerular nephritis; DG, Diabetic glomerulopathy; DKD, Diabetes kidney disease; FSGS, focal segmental glomerular sclerosis, LPTD, light chain proximal tubular disease. ^ The κ restricted LPTD was diagnosed by EM examination.

**Table S2. Immunofluorescence staining features of glomeruli in IgG4-RKD**

|  | IgG4-TIN | IgG4-TIN,  MN | IgG4-TIN,  MN, CrGN | IgG4-MN | IgG4-TIN,  CrGN | IgG4TIN,  DKD |
| --- | --- | --- | --- | --- | --- | --- |
| No. | 17 | 3 | 2 | 2 | 11 | 3 |
| Autoantibodies |  |  | 1 PLA_2_R |  | 8 ANCA |  |
| Clinical syndrome | 4 AKD  8 AKD/CKD  5 CKD | 1 AKD  2 AKD/CKD | 2 AKD/CKD | 2 CKD | 9 AKD  2 CKD | 1 AKD  1 AKD/CKD  1 CKD |
| AKD % | 70.6% | 100% | 100% | 0 | 81.8% | 66.7% |
| IgG | 4/17, 23.5% | 3/3, 100% | 2/2, 100% | 2/2, 100% | 3/11,27.3% | 2/3, 66.7% |
| C3 | 5/17, 29.4% | 3/3, 100% | 2/2, 100% | 1/2, 50% | 7/11,63.3% | 2/3, 66.7% |
| IgG1 | 1/17, 5.9% | 3/3, 100% | 2/2, 100% | 2/2, 100% | 3/11,27.3% | 0 |
| IgG2 | 0 | 3/3, 100% | 2/2, 100% | 2/2, 100% | 3/11,27.3% | 0 |
| IgG3 | 2/17, 11.8% | 2/3, 100% | 2/2, 100% | 0 | 3/11,27.3% | 0 |
| IgG4 | 1/17, 5.9% | 3/3, 100% | 2/2, 100% | 2/2, 100% | 4/11,36.4% | 0 |
| C1q | 3/17, 17.6% | 0 | 1/2, 50% | 0 | 1/11,9.1% | 2/3, 66.7% |
| Kappa | 0 | 0 | 2/2, 100% | 0 | 2/11,18.2% | 0 |
| Lambda | 2/17, 11.8% | 0 | 2/2, 100% | 0 | 3/11,27.3% | 0 |
| TBM deposit | 10/17, 58.8% | 3/3, 100% | 2/2, 100% | 0 | 1/11,9.1% | 1/3, 33.3% |
| Eosinophil infiltration | 16/17, 94.1% | 3/3, 100% | 2/2, 100% | 0 | 2/11,18.2% | 3/3, 100% |
| Storiform fibrosis | 11/17, 64.7% | 1/3, 33.3% | 0 | 0 | 3/11,27.3% | 1/3, 33.3% |
